# Combined Functional Assessment for Predicting Clinical Outcomes in Stroke Patients After Post-acute Care: A Retrospective Multi-Center Cohort in Central Taiwan

**DOI:** 10.3389/fnagi.2022.834273

**Published:** 2022-06-17

**Authors:** Shuo-Chun Weng, Chiann-Yi Hsu, Chiung-Chyi Shen, Jin-An Huang, Po-Lin Chen, Shih-Yi Lin

**Affiliations:** ^1^Department of Post-baccalaureate Medicine, College of Medicine, National Chung Hsing University, Taichung, Taiwan; ^2^Division of Nephrology, Department of Internal Medicine, Center for Geriatrics and Gerontology, Taichung Veterans General Hospital, Taichung, Taiwan; ^3^Institute of Clinical Medicine, School of Medicine, College of Medicine, National Yang Ming Chiao Tung University, Taipei, Taiwan; ^4^Biostatistics Task Force of Taichung Veterans General Hospital, Taichung, Taiwan; ^5^Neurological Institute, Taichung Veterans General Hospital, Taichung, Taiwan; ^6^Institute of Brain Science, School of Medicine, National Yang Ming Chiao Tung University, Taipei, Taiwan; ^7^Division of Endocrinology and Metabolism, Department of Internal Medicine, Center for Geriatrics and Gerontology, Taichung Veterans General Hospital, Taichung, Taiwan

**Keywords:** Berg Balance Scale, Fugl-Meyer Assessment, Functional Oral Intake Scale, post-acute care, mortality, readmission

## Abstract

**Background and Objective:**

In 2014, Taiwan’s National Health Insurance administration launched a post-acute care (PAC) program for patients to improve their functions after acute stroke. The present study was aimed to determine PAC assessment parameters, either alone or in combination, for predicting clinical outcomes.

**Methods:**

We retrospectively enrolled stroke adult patients through one PAC network in central Taiwan between January 2014 and December 2020. We collected data on post-stroke patients’ functional ability at baseline and after PAC stay. The comprehensive assessment included the following: Modified Rankin Scale (MRS), Functional Oral Intake Scale (FOIS), Mini-Nutritional Assessment (MNA), Berg Balance Scale (BBS), Fugl-Meyer Assessment (FMA), Mini-Mental State Examination (MMSE), aphasia test, and quality of life. The above items were assessed first at baseline and again at discharge from PAC. Logistic regression was used to determine factors that were associated with PAC length of stay (LOS), 14-day hospital readmission, and 1-year mortality.

**Results:**

A total of 267 adults (mean age 67.2 ± 14.7 years) with completed data were analyzed. MRS, activities of daily living (ADLs), instrumental activities of daily living (IADLs), BBS, and MMSE all had improved between disease onset and PAC discharge. Higher baseline and greater improvement of physical and cognitive functions between initial and final PAC assessments were significantly associated with less readmission, and lower mortality. Furthermore, the improved ADLs, FOIS, MNA, FMA-motor, and MMSE scores were related to LOS during PAC. Using logistic regression, we found that functional improvements ≥5 items [adjusted odds ratio (aOR) = 0.16; 95% confidence interval (CI) = 0.05–0.45] and improved MMSE (aOR = 0.19; 95% CI = 0.05–0.68) were significantly associated with reduced post-PAC mortality or readmission. Whereas, functional improvements ≥7 items, improved FOIS, and MNA significantly prolonged LOS during PAC.

**Conclusion:**

Physical performance parameters of patients with acute stroke improved after PAC. PAC assessment with multiple parameters better predicted clinical outcomes. These parameters could provide information on rehabilitation therapy for acute stroke patients receiving PAC.

## Introduction

The main purpose of post-acute care (PAC) is to achieve functional and occupational recovery and to maintain psychospiritual homeostasis after acute illness, especially on patients with rehabilitation potential but in advanced age and with multimorbidity (Pyrkov et al., 2021). Current guideline, from both the American Heart Association and American Stroke Association (Winstein et al., 2016), and the National Health Service in the United Kingdom, recommend that patients who are candidates for post-acute rehabilitation to receive individualized, interprofessional care, and multicomponent exercise intervention including high-intensity resistance training, to reverse functional decline after major diseases. To improve rehabilitation outcomes on neuromuscular performance (muscle strength and power), mobility, and spasticity, several interventions, including whole-body vibration exercise, mirror therapy, proprioceptive neuromuscular facilitation, and neuro-developmental technique also referred as the Bobath concept, have been used in patients after acute stroke (Liao et al., 2015; Guiu-Tula et al., 2017; Oliveira et al., 2018; Sañudo et al., 2018; Thieme et al., 2018; Díaz-Arribas et al., 2020). Based on the variable effectiveness of different techniques, knowledge for response prediction seems important in people with stroke to guide better individualized rehabilitation protocols.

In the report of U.S. Nursing Home Compare (Burke et al., 2021), the target of payment reforms or payment in the performance program for skilled nursing facility (SNF) was based on patient outcome in terms of successful community discharge rate (Burke et al., 2021). However, PAC should also target patient-oriented outcomes (e.g., 1-year mortality, 14-day or 30-day hospital readmission, and functional improvement) along with the growing concern regarding those elderly with poor resilience being “rehabbed to death” (Burke et al., 2021). The medical literature reported, on functional improvements of acute stroke patients, prognostic factors like the onset time of rehabilitation, duration and intensity of treatment, and the place of care (Vazquez-Guimaraens et al., 2021). To improve clinical outcomes after PAC, knowledge of their determinant factors is important. It was reported that a combination of the severity of deficits, cognitive ability, comorbidity, and response to objectives, can provide a more individualized treatment period, improving clinical outcomes, such as emergency room visit, hospital readmission, and mortality (Stinear et al., 2017; Vazquez-Guimaraens et al., 2021).

In Taiwan, the PAC network for patients with acute cerebrovascular accidents (CVA) started in January 2014 (Lee et al., 2014), and the National Health Insurance (NHI) covers costs up to a maximal 12 weeks of PAC-CVA when patients receive rehabilitation of physical, occupational, and speech therapy. Unlike multiple choices (SNFs, long-term care hospitals, inpatient rehabilitation, and home health services) for PAC settings in United States and United Kingdom (White, 2019), in Taiwan, most PAC settings are community hospital-based with reimbursement from the NHI for clinicians, physical, occupational specialists, and speech-language pathologists who have been well-trained with cross-regional working ability (Lee et al., 2014). Several studies reported the effectiveness of PAC in helping patients return home after a stroke, improving and accelerating functional recovery (Peng et al., 2017; Wang et al., 2017; Hsieh et al., 2018). In Taiwan’s PAC program several parameters are assessed, like Modified Rankin Scale (MRS), Functional Oral Intake Scale (FOIS), Berg Balance Scale (BBS), Fugl-Meyer Assessment (FMA), Mini-Nutritional Assessment (MNA), Mini-Mental State Examination (MMSE), Concise Chinese Aphasia Test (CCAT), and 3-level 5-dimensional Euro-Quality of Life (EQ-5D-3L). Initial functional status, disease severity, and balance status were reported to be associated with mortality in patients with acute stroke (Ho et al., 2014; Foroozanfar et al., 2020). Furthermore, cognitive impairment after stroke is related to poor clinical outcomes, such as a higher rate of disability, the institutionalization of long-term care facilities, recurrence of stroke, and mortality (Li J. et al., 2020). During hospitalization for stroke, premorbid undernutrition, and oropharyngeal dysphagia are known to have adverse effects on the prognosis of stroke patients by having greater complications, mortality, length of stay (LOS), and poor neurological outcomes (Kang et al., 2020; Souza et al., 2020). Prognosis of stroke reflects complex interactions of multiple risk factors, and that can be better judged through the construction of tools. Here, we hypothesized that by combining multiple clinical measures in the PAC program, those patients with a greater chance of recovery after rehabilitation can be identified. Consequently, individualized care planning can be made to improve patients’ clinical outcomes. To test the hypothesis, a multi-center cohort was conducted. We aimed to use comprehensive assessments in patients with acute CVA at the acute hospital and under PAC settings and to evaluate factors associated with clinical outcomes, including LOS at PAC, 14-day hospital readmission, and 1-year mortality.

## Materials and Methods

### Study Design

We used a retrospective longitudinal design to explore clinical relevant factors of PAC outcomes in acute stroke patients, including LOS at PAC, 14-day hospital readmission, and 1-year mortality. In reporting this study, the standard methodology was reported according to “Strengthening the Reporting of Observational Studies in Epidemiology” (STROBE) guidelines (Cuschieri, 2019). The study was approved by the Institutional Review Board of TCVGH (No. CE21441B).

### Setting

**FIGURE 1:**
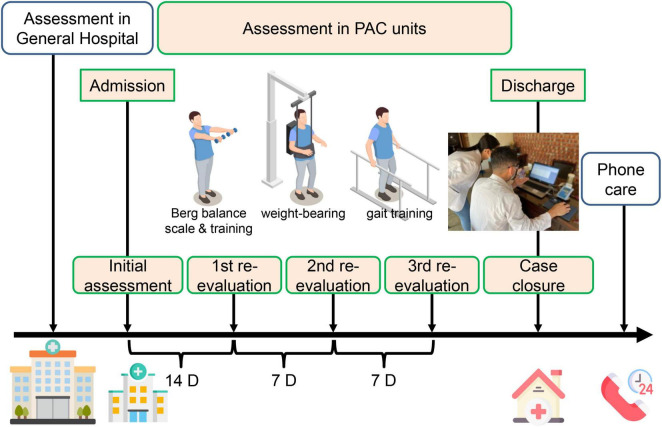
A simple schematic diagram illustrating the assessment and measurement protocol executed in the general hospital, followed by post-acute care units. PAC, post-acute care. Some images are edited by Freepik free website and software, https://www.freepik.com/home.

In this study, the PAC network (virtual private network, VPN) was created in January 2014 in our general hospital with 37 community hospital mastering PAC–stroke rehabilitation. Candidate patients in acute ward would first be evaluated by a case manager, and then transferred to PAC hospital for rehabilitation if they met the program criteria, where a hospital-based multidisciplinary team, composed of a physiatrist, physical therapist, occupational therapist, speech therapist, social worker, and case manager, managed the rehabilitation program with 1–3 h of intensive rehabilitation per workday over the following 6–12 weeks. A formal functional assessment was evaluated in those acute stroke participants before discharge in the acute ward of general hospital, and subsequently several times in the PAC hospital at admission, first re-evaluation 14 days later, second re-evaluation 7 days later, third re-evaluation 7 days later, and case closure (Figure 1).

### Study Participants

**FIGURE 2:**
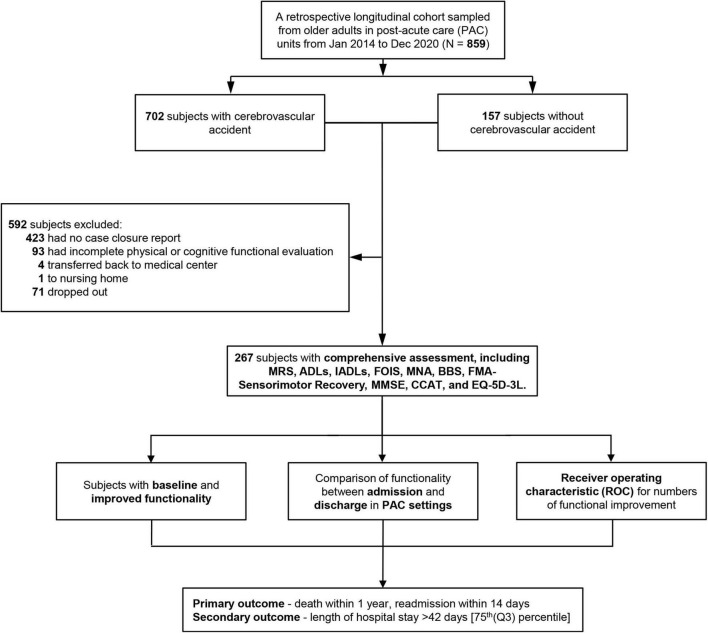
Flowchart of patient selection. MRS, Modified Rankin Scale; ADLs, activities of daily living; IADLs, instrumental activities of daily living; FOIS, Functional Oral Intake Scale; MNA, Mini-Nutritional Assessment; BBS, Berg Balance Scale; FMA, Fugl-Meyer Assessment; MMSE, Mini-Mental State Examination; CCAT, Concise Chinese Aphasia Test; EQ-5D-3L, 3-level 5-dimensional European Quality of Life questionnaire; PAC, post-acute care.

During the study period, patients with a primary diagnosis of acute stroke in our hospital, met the following criteria: (1) stroke onset time within 1 month, (2) stable hemodynamic parameters and no neurological deterioration within 72 h, and MRS between 2 and 4 (between 3 and 4 since July 2017 due to the policy change), and being transferred to PAC hospital with complete admission and discharge records, were enrolled. Those patients with (1) incomplete physical or cognitive assessment and (2) being transferred back to the medical center, or a nursing home before discharge were excluded. Overall, there were 702 patients with stroke who received the PAC program between January 2014 and December 2020. However, among those who were successfully transferred to a PAC institution, we excluded 423 patients due to no closure report, and 93 with incomplete data on their functional ability during PAC stay. Besides, four patients who ended their PAC due to hospitalization for acute illness, and one patient who was admitted to a nursing home due to non-stroke disease progression, were excluded. Finally, 267 post-stroke patients who completed their PAC stay and for whom complete data on functional ability were available were enrolled in our study (Figure 2).

### Assessments of Stroke Severity and Functional Abilities

Baseline characteristics of patients including index data in the general hospital and PAC community hospital were collected. Besides, medical staff routinely conducted a minimum of 10 measurements on a patient with CVA in the multiple PAC settings (Figure 2). The MRS was used to measure stroke severity and handicap (Burn, 1992). The Barthel Index was used to evaluate ADLs, and the Lawton scale was used to evaluate IADLs (Lawton and Brody, 1969; Collin et al., 1988). The MNA and MMSE were used to identify CVA patients at risk for malnutrition and memory impairment (Guigoz, 2006). The FOIS (Crary et al., 2005) was recorded with a score of 1 [(worse) nothing by mouth] to 7 [(best) complete oral diet with no restrictions]. The interrater reliability was determined with six speech-language pathologists. The total scale and subscales of the BBS were further used to evaluate the functional independence of patients with CVA (Cheng et al., 2014). Both upper extremity motor subscore (0–66) and modified sensation (range of joint motion; 0–44) of the FMA (five domains: motor function, sensory function, balance, joint range of motion, joint pain) were used as predictors of poor functional recovery after sensorimotor stroke (Sullivan et al., 2011). For all sensory and motor assessments, we found high consistency between the therapist raters and expert raters among PAC settings. Language performance was assessed against all language modalities with the CCAT designed for standardized, linguistically, culturally neutral, and native Mandarin Chinese speakers (Tsai et al., 2014). We evaluated four subcategories of CCAT related to language production. They were conversation, family picture description, object naming and expression, and repetition. For assessment of the quality of life, we used the EQ-5D-3L tool (mobility, self-care, usual activities, pain/discomfort, and anxiety/depression) (Lin et al., 2020).

### Outcome Measurements and Factor Analyses

A primary clinical outcome was 1-year mortality or 14-day unplanned hospital readmission. Information on all-cause mortality was obtained from the PAC network, with validation in Taiwan’s National Death Registry, according to the ICD-9 (ICD9 001.x-999.x) or ICD10 (A00.x-Z99.x). LOSs in the PAC hospital were stratified into four quartiles, and LOS exceeding 42 days [the third quartile (Q3)] was defined as a secondary outcome.

To allow better prediction of clinical outcomes, various combinations of improved functional parameters were analyzed. Functional improvement at the end of PAC training was defined by improved scores of MRS, ADLs, IADLs, FOIS, MNA, BBS, FMA-motor, FMA-modified sensation, MMSE, and CCAT (Lai et al., 2017).

### Statistical Analyses

For continuous variables, we first used the Kolmogorov–Smirnov test to determine the normality of sample distribution. Continuous variables were then analyzed with the Mann–Whitney *U* test, generating the median and interquartile range (IQR). Categorical variables, expressed as percentages, were tested with Chi-square or Fisher’s exact test. We used Wilcoxon signed-rank test, a non-parametric test, to test the location of a set of samples or to compare locations of two populations using a set of matched samples. Both primary and secondary outcomes were correlated and delineated based on previously defined functional status in general hospitals and community PAC hospitals, regarding demography and laboratory test results. Logistic regression analyses were finally applied. Specifically, we used multivariate analyses to estimate odds ratios (ORs) of outcomes after adjusting for age, gender, body mass index (BMI), and cardiovascular disease (CVD). Besides, to test the discrimination ability of the item numbers of improved functionality on clinical outcome, a cut-off value was determined by the area under the ROC curve (AUC) under the non-parametric assumption. *p*-Values for non-linearity were calculated using the null hypothesis test. Statistical significance was set at *p* < 0.05. All analyses were performed with the SPSS for Windows version 22.0 (SPSS Institute Inc., Chicago, IL, United States).

## Results

The median age of 267 CVA patients was 67.0 years (IQR = 58.0–79.0), 32.2% with smoking behaviors, 17.6% with alcohol consumption. Their median BMI was 24.4 kg/m^2^ (IQR = 22.1–27.3), 36.3% had diabetes mellitus, 84.3% with hypertension, median LDL-c was 100.0 mg/dL (IQR = 85.0–125.0), fasting glucose was 123 mg/dL (IQR = 105.0–159.0), and glycated hemoglobin (Hba1c) was 6.0% (IQR = 5.6–6.6) (Table 1). Among patients with CVA, median MRS was 4.0 (IQR = 4.0–4.0), ADLs was 35.0 (IQR = 20.0–55.0), and IADLs was 1.0 (IQR = 0.0–2.0). They showed moderately severe disability and were unable to walk and unable to attend to bodily needs without assistance. FOIS with a median of 6.0 (IQR = 3.0–6.0) meant that CVA patients suffered from nasogastric tube dependent on the consistent oral intake of food or liquid to total oral diet with multiple consistencies without special preparation, but with specific food limitations. EQ-5D-3L, a multi-dimensional term, showed an average sub-score of 2.0–3.0, reflecting that these patients were not satisfied with their physical, social, and emotional well-being.

**TABLE 1:** Baseline characteristics of patients with cerebrovascular accident.

Cerebrovascular accident	*n* = 267
Ischemia (%)	222 (83.1)
Hemorrhage (%)	45 (16.9)
**Demographic profile**	
Age, median (IQR, years)	67.0 (58.0–79.0)
Gender, male (%)	172 (64.4)
Smoking (%)	86 (32.2)
Alcohol (%)	47 (17.6)
BMI, median (IQR, kg/m^2^)	24.4 (22.1–27.3)
**Comorbidity profile (%)**	
Diabetes mellitus	97 (36.3)
Hypertension	225 (84.3)
Hyperlipidemia	159 (59.6)
Cardiovascular disease	106 (39.7)
COPD	7 (2.6)
ACCI, median (IQR)	4.0 (3.0–6.0)
**Laboratory profile, median (IQR)**	
Low-density lipoprotein cholesterol (mg/dL)	100.0 (85.0–125.0)
Fasting glucose (mg/dL)	123.0 (105.0–159.0)
Hba1c (%)	6.0 (5.6–6.6)
Albumin (g/dL)	3.9 (3.6–4.1)
eGFR (mL/min per 1.73 m^2^)	82.9 (61.6–103.8)
Urine protein/creatinine ratio (mg/g)	0.1 (0.1–0.3)
NT-proBNP (pg/mL)	1333.0 (235.0–3450.0)
**Assessment in general hospital, median (IQR)**	
Modified Rankin Scale	4.0 (4.0–4.0)
ADLs	35.0 (20.0–55.0)
IADLs	1.0 (0.0–2.0)
Functional Oral Intake Scale	6.0 (3.0–6.0)
Mini-Nutritional Assessment	18.5 (14.5–21.0)
**EQ-5D-3L**	
Mobility	2.0 (2.0–3.0)
Self-care	3.0 (2.0–3.0)
Usual activities	3.0 (2.0–3.0)
Pain/discomfort	2.0 (1.0–2.0)
Anxiety/depression	2.0 (1.0–2.0)

*Continuous data were expressed as median (IQR, interquartile range) and analyzed by the Kruskal–Wallis test. Categorical data were expressed as number and percentage and analyzed by the Chi-square test.*

*BMI, body mass index; COPD, chronic obstructive pulmonary disease; ACCI, age-adjusted Charlson Comorbidity Index; eGFR, estimated glomerular filtration rate; NT-proBNP, N-terminal pro-B-type natriuretic peptide; ADLs, activities of daily living; IADLs, instrumental activities of daily living; EQ-5D-3L, 3-level 5-dimensional European Quality of Life questionnaire; eGFR, calculated by using modified modification diet of renal disease (MDRD) formula, was utilized to evaluate renal function.*

When initial values were compared with those after this program in the PAC settings, we found significant improvements in all functionalities, including MRS, ADLs, IADLs, FOIS, MNA, BBS, FMA, MMSE, CCAT, and EQ-5D-3L (mobility, self-care, usual activities, pain/discomfort, and anxiety/depression) (Table 2). At enrollment in the PAC settings, 16 patients (6.0%) were classified as MRS 2, 59 patients (22.1%) as MRS 3, and 190 (71.2%) patients as MRS 4 (Supplementary Table 1). Upon case closure, 143 patients (53.6%) showed improved MRS levels by scores ≥1. The difference in ADL scores before and after hospitalization showed improvements in 227 patients (85.0%). The score of FOIS improved in 108 patients (40.4%) and similarly BBS scores improved in 231 patients (86.5%). Table 2 shows changes of EQ-5D-3L.

**TABLE 2:** Effect of PAC on functional performance and quality of life in patients with stroke.

	Admission	Discharge	*p*-Value
MRS	4.0 (3.0–4.0)	3.0 (2.0–4.0)	<0.001
ADLs	40.0 (20.0–65.0)	70.0 (50.0–90.0)	<0.001
IADLs	1.0 (0.0–2.0)	2.0 (1.0–4.0)	<0.001
FOIS	6.0 (5.0–7.0)	7.0 (6.0–7.0)	<0.001
MNA	18.3 (14.0–22.5)	21.0 (16.5–24.0)	<0.001
BBS	20.0 (4.0–38.0)	40.5 (19.0–51.0)	<0.001
FMA-modified sensation	39.0 (24.0–44.0)	44.0 (36.0–44.0)	<0.001
FMA-motor	45.0 (15.5–59.0)	57.0 (33.5–62.0)	<0.001
MMSE	20.0 (12.0–26.0)	25.0 (19.0–29.0)	<0.001
CCAT	10.5 (7.3–11.5)	11.0 (8.9–11.9)	<0.001
**EQ-5D-3L**			
Mobility	2.0 (2.0–2.0)	2.0 (1.0–2.0)	<0.001
Self-care	2.0 (2.0–3.0)	2.0 (1.0–2.0)	<0.001
Usual activities	2.0 (2.0–3.0)	2.0 (2.0–2.0)	<0.001
Pain/discomfort	2.0 (1.0–2.0)	1.0 (1.0–2.0)	<0.001
Anxiety/depression	2.0 (1.0–2.0)	1.0 (1.0–2.0)	<0.001

*Continuous data were expressed as median (IQR, interquartile range) and analyzed by the Wilcoxon signed ranks test.*

*MRS, Modified Rankin Scale; ADLs, activities of daily living; IADLs, instrumental activities of daily living; FOIS, Functional Oral Intake Scale; MNA, Mini-Nutritional Assessment; BBS, Berg Balance Scale; FMA, Fugl-Meyer Assessment; MMSE, Mini-Mental State Examination; CCAT, Concise Chinese Aphasia Test; EQ-5D-3L, 3-level 5-dimensional European Quality of Life questionnaire.*

The functional changes of patients at baseline and discharge from the PAC hospital were shown in Table 2. The mean MRS score was 3.7 (SD 0.6) at admission, and that improved to 2.9 (SD 1.0) at discharge. The ADL scores improved from 43.2 (SD 26.3) at admission to 66.2 (SD 27.5) at discharge; IADLs also improved from 1.5 (SD 1.6) to 2.5 (SD 2.1). Similarly, the nutritional status measured by FOIS improved from 5.4 (SD 2.0) to 6.1 (SD 1.7), and by MNA improved from 18.0 (SD 5.1) to 19.8 (SD 5.3). In the balance domain, BBS improved from 21.9 (SD 18.2) before PAC training to 34.7 (SD 17.9) at discharge. In the mental status, the MMSE score improved from 18.6 (SD 9.0) initially to 22.2 (SD 8.5) at discharge. In the language aspect, the CCAT score also improved from 8.9 (SD 3.6) to 9.4 (SD 3.4). In the EQ-5D-3L dimension of the generic health status of the significant improvements were observed for all subcategories at discharge, including mobility [from 2.2 (SD 0.6) to 1.8 (SD 0.6), *p* < 0.001], self-care [from 2.3 (SD 0.6) to 1.8 (SD 0.6), *p* < 0.001], usual activity [from 2.3 (SD 0.6) to 2.0 (SD 0.7), *p* < 0.001], pain/discomfort [from 1.5 (SD 0.6) to 1.4 (SD 0.5), *p* < 0.001], and anxiety/depression [from 1.6 (SD 0.6) to 1.4 (SD 0.5), *p* < 0.001]. For the pain/discomfort and anxiety/depression subcategories, changes occurred predominantly in terms of a shift in the median from level 2 to level 1 (*p* < 0.001).

**FIGURE 3:**
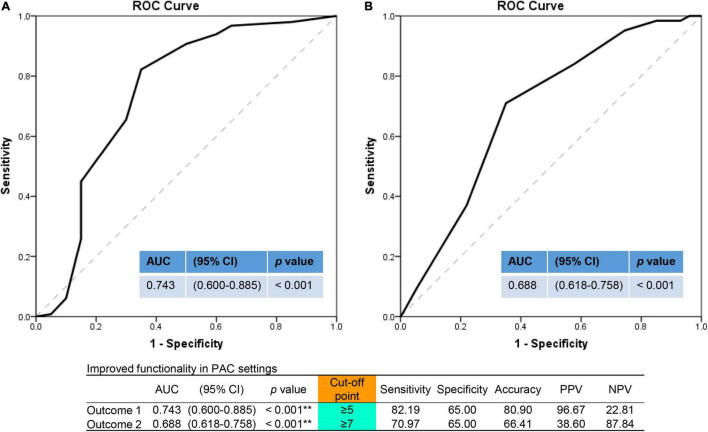
Outcome prediction assessed by numbers of improved functionality items in the area under the ROC curve (AUC) analysis. **(A)** For primary outcome (1-year mortality or 14-day readmission). **(B)** For secondary outcome (length of hospital stay >42 days). ROC, receiver operating characteristic curve; AUC, area under curve; PAC, post-acute care; CI, confidence interval; PPV, positive predictive value; NPV, negative predictive value. **p < 0.01.

Cerebrovascular accident patients with 1-year mortality or 14-day hospital readmission had a higher percentage of ischemic stroke (90.0%), of older age [median 78.0 years (IQR = 66.3–84.0)], with lower BMI of 22.2 kg/m^2^ (IQR = 19.7–24.9), higher CVD (75%), small numbers of improved functionality with a median 3.5 items (IQR = 1.0–6.0) vs. 6.0 items (IQR = 5.0–8.0), lower percentages of improved MRS, ADLs, IADLs, BBS, MMSE, and lower levels of improved EQ-5D-3L (Supplementary Table 2). Further, it was shown that an optimal cut-off values of improved PAC items to predict primary outcome was 5 with an AUC of 0.74 [95% confidence interval (CI) = 0.60–0.89, sensitivity: 82.2%, specificity: 65.0%, *p* < 0.001], and 7 for second outcome (LOS >42 days) with an AUC of 0.69 (95% CI = 0.62–0.76, sensitivity: 71.0%, specificity: 65.0%, *p* < 0.001) (Figure 3).

In the simple logistic regression, CVA patients with older age and CVD were significantly associated with increased risk of 1-year mortality or 14-day hospital readmission. On the other hand, higher BMI, higher numbers of improved function, improved functional number greater or equal to 5, improved MRS, ADLs, IADLs, BBS, and MMSE were all significantly associated with reduced risks of the primary outcome (Table 3). In the multivariate logistic regression model, improved functional number per item [model 1, Table 3, adjusted OR (aOR) = 0.69; 95% CI = 0.55–0.86; *p* = 0.001], improved functional numbers (≥5 vs. <5, model 2, Table 3, aOR = 0.16; 95% CI = 0.05–0.45; *p* = 0.001), and high MMSE (model 3, Table 3, aOR = 0.19; 95% CI = 0.05–0.68; *p* = 0.010) were significantly associated with reduced all-cause mortality or 14-day hospital readmission after adjusting for age, BMI, CVD, and different items of improved functionality.

**TABLE 3:** Predictors of 1-year mortality or 14-day readmission outcome in patients with PAC.

	Simple model	Model 1	Model 2	Model 3
				
	OR (95% CI)	OR (95% CI)	OR (95% CI)	OR (95% CI)
Age	1.05 (1.01–1.09)*	1.01 (0.98–1.05)	1.01 (0.97–1.05)	1.03 (0.98–1.08)
Female vs. male	0.76 (0.28–2.05)			
BMI	0.77 (0.66–0.90)**	0.74 (0.62–0.89)**	0.74 (0.62–0.89)**	0.70 (0.57–0.87)**
Cardiovascular disease	5.14 (1.81–14.62)**	4.90 (1.46–16.41)*	6.36 (1.82–22.19)**	3.53 (0.94–13.33)
ACCI	1.16 (0.95–1.42)			
Improved functional numbers	0.65 (0.54–0.79)**	0.69 (0.55–0.86)**		
Improved functional numbers (≥5 vs. <5)	0.12 (0.04–0.31)**		0.16 (0.05–0.45)**	
**Improved functionality**				
MRS	0.19 (0.06–0.60)**			0.73 (0.17–3.14)
ADLs	0.11 (0.04–0.28)**			0.52 (0.12–2.28)
IADLs	0.28 (0.09–0.89)*			0.66 (0.16–2.69)
FOIS	0.98 (0.39–2.48)			
MNA	0.47 (0.18–1.21)			
BBS	0.22 (0.08–0.61)**			0.46 (0.10–2.11)
FMA-modified sensation	0.74 (0.27–2.00)			
FMA-motor	0.53 (0.19–1.50)			
MMSE	0.25 (0.09–0.68)**			0.19 (0.05–0.68)*
CCAT	0.35 (0.07–1.71)			

*p < 0.05; **p < 0.001; Model 1: the logistic regression was used to evaluate the association of primary outcome with multivariate analysis among age, body mass index (BMI), cardiovascular disease (CVD), and numbers of functional improvement in patients with post-acute care (PAC). Model 2: the logistic regression was used to evaluate the association of primary outcome with multivariate analysis among age, BMI, CVD, and categorized functional improvement in patients with PAC.

*Model 3: the logistic regression was used to evaluate the association of primary outcome with multivariate analysis among age, BMI, CVD, and different functional improvement in patients with PAC.*

*ACCI, age-adjusted Charlson Comorbidity Index; MRS, Modified Rankin Scale; ADLs, activities of daily living; IADLs, instrumental activities of daily living; FOIS, Functional Oral Intake Scale; MNA, Mini-Nutritional Assessment; BBS, Berg Balance Scale; FMA, Fugl-Meyer Assessment; MMSE, Mini-Mental State Examination; CCAT, Concise Chinese Aphasia Test.*

Some CVA patients with prolonged LOS were female gender (46.8%), had lower serum N-terminal pro-B-type natriuretic peptide, higher disability [median MRS 4 (IQR = 4–4)], lower ADLs 25.0 (IQR = 15.0–40.0), lower IADLs 0.0 (IQR = 0.0–1.0), poorer FOIS 5.0 (IQR = 2.0–6.0), and lower MNA 16.5 (IQR = 13.0–19.8) in general hospitals. They had, however, a greater improved functional number with 71.0% ≥7, higher percentages of improved ADLs, FOIS, MNA, FMA-motor, MMSE, CCAT, and higher levels of improved anxiety/depression in EQ-5D-3L (Supplementary Table 3). In the multivariate logistic regression model in CVA patients within PAC units, improved functional number per item (model 1, Table 4, aOR = 1.46; 95% CI = 1.22–1.75; *p* < 0.001), improved functional numbers (≥7 vs. <7, model 2, Table 4, aOR = 4.73; 95% CI = 2.48–9.02; *p* < 0.001), higher FOIS (model 3, Table 4, aOR = 2.33; 95% CI = 1.21–4.47; *p* = 0.011), and high MNA (model 3, Table 4, aOR = 3.51; 95% CI = 1.44–8.56; *p* = 0.006) were significantly associated with prolonged LOS after adjusting for age, gender, and different items of improved functionality. But in the multivariate logistic regression model on CVA patients with assessments in general hospitals, higher baseline FOIS (model 5, Table 4, aOR = 0.81; 95% CI = 0.66–0.999, *p* = 0.049) was significantly associated with reduced LOS after adjusting for age, gender, and different items of functionality.

**TABLE 4:** Predictors of length of stay in patients with PAC.

	Simple mode	Model 1	Model 2	Model 3	Model 4
					
	OR (95% CI)	OR (95% CI)	OR (95% CI)	OR (95% CI)	OR (95% CI)
Age	1.00 (0.98–1.02)	1.01 (0.99–1.03)	1.01 (0.99–1.03)	1.01 (0.98–1.03)	1.00 (0.98–1.03)
Female vs. male	1.91 (1.07–3.42)*	1.67 (0.90–3.07)	1.67 (0.90–3.10)	1.72 (0.88–3.36)	1.86 (0.84–4.13)
BMI	1.02 (0.94–1.10)				
**Assessment in general hospital**					
MRS	3.46 (1.50–7.96)**				2.21 (0.69–7.03)
ADLs	0.97 (0.96–0.99)**				1.00 (0.97–1.03)
IADLs	0.71 (0.53–0.95)*				0.86 (0.60–1.23)
FOIS	0.84 (0.73–0.96)*				0.81 (0.66–0.999)*
MNA	0.91 (0.86–0.97)**				0.97 (0.88–1.06)
Improved functional numbers	1.46 (1.22–1.75)**	1.46 (1.22–1.75)**			1.49 (1.17–1.89)**
Improved functional numbers (≥7 vs. <7)	4.54 (2.44–8.44)**		4.73 (2.48–9.02)**		
**Improved functionality**					
MRS	1.03 (0.58–1.83)				
ADLs	7.04 (1.65–30.07)**			5.60 (0.69–45.24)	
IADLs	1.30 (0.73–2.33)				
FOIS	3.12 (1.72–5.68)**			2.33 (1.21–4.47)*	
MNA	3.57 (1.71–7.43)**			3.51 (1.44–8.56)**	
BBS	1.53 (0.60–3.88)				
FMA-modified sensation	1.61 (0.89–2.88)				
FMA-motor	2.72 (1.16–6.38)*			1.83 (0.68–4.90)	
MMSE	2.41 (1.07–5.42)*			1.80 (0.74–4.38)	
CCAT	1.68 (0.78–3.63)				

*p < 0.05; **p < 0.001; Model 1: the logistic regression was used to evaluate the association of secondary outcome with multivariate analysis among age, gender, and numbers of functional improvement in patients with post-acute care (PAC). Model 2: the logistic regression was used to evaluate the association of secondary outcome with multivariate analysis among age, gender, and categorized functional improvement in patients with PAC. Model 3: the logistic regression was used to evaluate the association of secondary outcome with multivariate analysis among age, gender, and different functional improvement in patients with PAC in the PAC units. Model 4: the logistic regression was used to evaluate the association of secondary outcome with multivariate analysis among age, gender, and different baseline functional assessment of patients in the general hospital.

*MRS, Modified Rankin Scale; ADLs, activities of daily living; IADLs, instrumental activities of daily living; FOIS, Functional Oral Intake Scale; MNA, Mini-Nutritional Assessment; BBS, Berg Balance Scale; FMA, Fugl-Meyer Assessment; MMSE, Mini-Mental State Examination; CCAT, Concise Chinese Aphasia Test.*

## Discussion

Our principal findings of this retrospective, multi-center cohort of patients with CVA are MMSE being higher after PAC intervention, and that was significantly associated with reduced 1-year mortality or 14-day unplanned hospital readmission. Besides, greater functional improvements, especially ≥5 items, were associated more with the primary outcome. The mortality and 14-day readmission were typically high amongst individuals with low BMI and CVD. Finally, prolonged LOS in PAC settings was correlated with improved FOIS and MNA in the PAC units, whereas better baseline FOIS in the general hospital was significantly associated with reduced LOS.

Post-acute care services are well developed in the United Kingdom and United States, likely to reduce hospital readmission. Taiwan has only started to establish PAC services in 2014 from intermediate care, interchangeable with PAC. The first community hospital-based PAC program was launched in 2007 (Chen et al., 2010), and that was later extended to five major diseases, including CVA, burn injury, fragile fracture, traumatic nerve injury, and frailty. Compatible with several previous reports in Taiwan (Lai et al., 2017; Peng et al., 2017, 2019; Chien et al., 2020), our study provided additional evidence that intervention of the PAC program significantly had improved stroke patients regarding their general condition (MRS), ADLs, IADLs, nutritional status, quality of life, intake condition, balance, speech function, and mental status (MMSE).

In our study, the 1-year mortality rate was 3.7% which was lower than the 30% as reported in the literature (Mar et al., 2015). Age, gender, stroke type, stroke severity, comorbidities, rehabilitation, and social status are known predictors of stroke mortality (Chen et al., 2019). In line with other reports on early stroke disability and long-term mortality (Ganesh et al., 2017; Chen et al., 2019), we also found that baseline and improved MRS scores had predicted the 1-year mortality. Findings highlighted the importance of measuring stroke functional outcomes and suggested that reducing early disability lowers long-term mortality.

The AUC of the ROC curve of the 100-Barthel index score ≥48 for prolonged PAC hospital stay was 0.688 (95% CI = 0.617–0.759, *p* < 0.001). This showed its predictive value in the prognosis of acute CVA patients, a finding that is consistent with previous studies (Wade and Hewer, 1987; Martinsson and Eksborg, 2006; Lee et al., 2013; Li Q. X. et al., 2020). Results above suggest that the mortality of stroke patients is closely related to physical dependence, and that better basic care should be provided to stroke patients to reduce their mortality especially within a year after stroke onset. However, if the scale evaluation of daily activity function turns out to be unstable, prospective studies for several days or longer are needed to verify the findings.

Due to inactivity, diseases could worsen, and the ability of body balance often is impaired among older people. The impairment might lead to dramatic consequences such as dependency on ADLs, admission to nursing homes, falls and fractures, and even mortality (Conradsson et al., 2007; Keevil et al., 2018). We found that the BBS in the death group was significantly different from that in the survival group. The AUC of the ROC curve of the BBS scoring ≥37 for prolonged PAC hospital stay was 0.637 (95% CI = 0.562–0.711, *p* = 0.001). This showed its predictive value in the prognosis of acute stroke patients and suggested that training of static and dynamic balance control is of great importance in rehabilitation for old people.

After a stroke, cognitive impairment is common, ranging from 11.6 to 56.3% among hospital-based studies (Patel et al., 2002; Zietemann et al., 2018). Pre-stroke, any post-stroke, and new-onset post-stroke dementia is consistently related to poor survival. Cognitive impairment as measured by MMSE is associated with impaired survival up to 4-year follow-up, although two shorter studies of 1-year reported that MMSE is not an independent predictor of survival (Patel et al., 2002; Zietemann et al., 2018). Our present findings are in line with those findings on the association of global MMSE with survival. This means that patients with mild stroke executive dysfunction might be present. Even motor difficulties and management of basic daily tasks are minimal. Patients may have more difficulty performing complex activities. Therefore, accurate and appropriately timed assessment is essential. Related to this, we found that improvement of MRS, balance test, ADLs/IADLs, and MMSE scores were associated with reduced readmission risk.

In our study, the mortality in stroke patients with CVD was higher than in previous reports. We also found that those with lower BMI had higher 1-year mortality, whereas those with a higher BMI had lower 1-year mortality. Our result is consistent with a previous follow-up study (Ryu et al., 2011), in which the all-cause mortality rate is inversely related to BMI in patients with ischemic stroke. Low baseline BMI could reflect low muscle mass and malnutrition (Kimura et al., 2017), of which are detrimental to stroke patients (Kawase et al., 2017). Stroke patients with low BMI at admission might also not recover well with general hospital diets. They are therefore more likely to show post-stroke complications. It was thus suggested that underweight ischemic stroke patients should receive appropriate management, including nutrition and rehabilitation.

In our study, the 14-day readmission rate in acute stroke patients after the PAC program was 4.9%. This proportion created a significant burden on the healthcare system, and that is an important target for quality improvement efforts (Lichtman et al., 2010; Wen et al., 2018; Keawpugdee et al., 2021). Known common causes of readmission are their age and comorbid history of coronary heart disease, heart failure, renal disease, respiratory disease, peripheral arterial disease, and diabetes. Regarding stroke-related factors, LOS was associated with a higher readmission rate, followed by bowel incontinence, feeding tube, and urinary catheter (Rao et al., 2016). In addition, social, financial factors, discharge location/need for nursing care, advanced age, poor post-stroke physical functioning also contributed to readmission risk in these patients (Lichtman et al., 2010).

As improved MRS, ADLs, IADLs, BBS, and MMSE can all reduce the risk of readmission, a routine plan with a continuous rehabilitation process is needed before discharge. Many factors influence subacute rehabilitation LOS, including stroke severity measured with the National Institute of Health Stroke Scale (NIHSS), ability to perform ADLs, or admission Functional Independence Measure (FIM) score (Garcia-Rudolph et al., 2020). This study showed that in the general hospital setting, CVA patients with higher baseline MRS, lower ADLs/IADLs, poorer FOIS, and MNA had longer LOS in PAC units. It was speculated that the more severe dysfunction was, the more complicated the rehabilitation process became, thus prolonging LOS. However, it should be noted that the greater the PAC LOS, the greater the number of improved rehabilitation assessments. The situation is similar to a previous study in Taiwan showing post-stroke patients having longer stay in a PAC institution have superior ADL function, balance and coordination, walking speed, and upper-limb dexterity and sensory function (Tung et al., 2021). Despite an upper limit in the PAC payment for LOS, it was suggested that the disability status of patients should be taken into account in planning PAC with preset rehabilitation goals (Tan et al., 2009, 2010).

Our findings that for prognostic prediction, larger numbers of improved assessment tools had discriminating power better than a single parameter are likely related to the following issues. First, patients with varying severity, different stroke subtype, and risk factors experience different recovery courses that plateau at different levels and times. This may limit the clinical utility of predictiveness in cases based on one characteristic variable at a specific time point, such as stroke severity at the onset of stroke. Therefore, using a combined patient-specific predictive model, one can generate individualized recovery profiles, assisting in tailored discharge planning for stroke survivors. Overall, we used a comprehensive statistical approach for data analysis to demonstrate the relationship between various rehabilitation tools and outcomes after PAC program in acute stroke patients for predicting clinical outcomes. Such prognostic information is important for clinicians, stroke survivors, and their carers.

There were several limitations in this study. First, this was a retrospective and single medical center-based study which would limit the generalization of results to the whole Taiwanese or the global population. Second, this study involved many PAC institutions, and some data bias can be introduced. For example, data were recorded by different personnel, and the efficacy of training was likely non-uniform among participants. Third, not all comorbidities were recorded, and thus only some possible factors were analyzed. Finally, the measurement scales used in our PAC might have inherent limitations. For example, the 6-min walking test cannot represent cardiopulmonary capacity in those bedridden patients. However, despite this, by combining various rehabilitation parameters, mortality and readmission in patients with stroke can be better predicted.

## Conclusion

The PAC rehabilitation unit was beneficial for patients with acute strokes in terms of not only improvement in ADL function but also in the quality of life and balance function. Higher baseline and greater improvement of physical and cognitive function were significantly associated with less 1-year mortality and 14-day hospital readmission. The improved ADLs, FOIS, MNA, FMA-motor, and MMSE scores were related to LOS during PAC. Through classification by combined functional assessments, clinical outcomes can be better predicted. It is suggested that stroke patients with such conditions require better intensive care and risk-factor control at deciding discharge times for outcome improvements.

## Data Availability Statement

The original contributions presented in the study are included in the article/Supplementary Material, further inquiries can be directed to the corresponding author.

## Ethics Statement

The studies involving human participants were reviewed and approved by the Institutional Review Board of Taichung Veterans General Hospital (No. CE21441B). The patients/participants provided their written informed consent to participate in this study.

## Author Contributions

S-CW: conceptualization, investigation, formal analysis, and writing – original draft. C-YH: data curation, formal analysis, and investigation. C-CS, J-AH, and P-LC: conceptualization, investigation, and funding acquisition. S-YL: conceptualization, investigation, formal analysis, writing – review and editing, and supervision. All authors reviewed and approved the manuscript prior to submission.

## Conflict of Interest

The authors declare that the research was conducted in the absence of any commercial or financial relationships that could be construed as a potential conflict of interest.

## Publisher’s Note

All claims expressed in this article are solely those of the authors and do not necessarily represent those of their affiliated organizations, or those of the publisher, the editors and the reviewers. Any product that may be evaluated in this article, or claim that may be made by its manufacturer, is not guaranteed or endorsed by the publisher.
