# Supplementary material for: Combined Functional Assessment for Predicting Clinical Outcomes in Stroke Patients After Post-acute Care: A Retrospective Multi-Center Cohort in Central Taiwan
Source: Front Aging Neurosci. 2022 Jun 17;14:834273. doi: 10.3389/fnagi.2022.834273 (PMC9247545; doi:10.3389/fnagi.2022.834273)
Supplement: Supplementary file 1 [file Data_Sheet_1.pdf]

Combined Functional Assessment for Predicting Clinical Outcomes in Stroke Patients After Post-Acute Care: A Retrospective Multi-Center Cohort in Central Taiwan

Shuo-Chun Weng [1,2,3]*, Chiann-Yi Hsu*[4]*, Chiung-Chyi Shen*[5]*, Jin-An Huang*[5]*, Po-Lin Chen*[5,6] *and Shih-Yi Lin*[3,7*]

Supplementary Table 1 Effect of post-acute care (PAC) on Modified Rankin Scale (MRS) in patients with stroke

MRS	Admission	Discharge	*p* value
	n(%)	*n*(%)	
0	0(0.0)	1(0.4)	<0.001
1	1(0.4)	30(11.2)	
2	16(6.0)	49(18.4)	
3	59(22.1)	92(34.5)	
4	190(71.2)	95(35.6)	
5	1(0.4)	0(0.0)	

Supplementary Table 2 Primary outcome in patients with cerebrovascular events

	No (*n* = 247)	Yes (*n* = 20)	*p* value
**Cerebrovascular accident**			0.001
Ischemia (%)	204(82.6)	18(90.0)	
Hemorrhage (%)	43(17.4)	2(10.0)	
**Demographic profile**			
Age, median (IQR, years)	67.0(57.0-79.0)	78.0(66.3-84.0)	0.010
Gender, male (%)	158(64.0)	14(70.0)	0.765
Smoking (%)	77(31.2)	9(45.0)	0.306
Alcohol (%)[f]	44(17.8)	3(15.0)	1.000
BMI, median (IQR, kg/m^2)	24.6(22.2-27.5)	22.2(19.7-24.9)	0.001
**Comorbidity profile** (%)			
Diabetes Mellitus	89(36.0)	8(40.0)	0.910
Hypertension[f]	207(83.8)	18(90.0)	0.749
Hyperlipidemia	147(59.5)	12(60.0)	1.000
Cardiovascular disease	91(36.8)	15(75.0)	0.002
COPD[f]	7(2.8)	0(0.0)	1.000
ACCI, median (IQR)	4.0(3.0-6.0)	5.0(3.0-5.8)	0.255
**Laboratory profile**, median (IQR)			
Low-density lipoprotein cholesterol (mg/dL)	100.0(85.0-128.0)	99.5(88.3-115.0)	0.485
Fasting glucose (mg/dL)	123.0(105.0-161.0)	121.5(109.0-155.8)	0.890
Hba1c (%)	6.0(5.6-6.7)	5.8(5.5-6.6)	0.359
Albumin (g/dL)	3.9(3.6-4.1)	3.9(3.4-4.0)	0.353
eGFR (ml/min per $1.73m^2$)	83.6(61.6-104.0)	78.6(52.7-100.5)	0.469
Urine protien / creatinine ratio (mg/g)	0.1(0.1-0.3)	0.1(0.1-0.6)	0.739
NT-proBNP (pg/mL)	1460.0(235.0-3450.0)	380.5(254.5-9753.8)	0.604
**Assessment in General Hospital**, median (IQR)			
Modified rankin scale	4.0(4.0-4.0)	4.0(4.0-4.0)	0.686
ADLs	35.0(20.0-55.0)	32.5(17.5-40.0)	0.469
IADLs	1.0(0.0-2.0)	0.0(0.0-1.8)	0.572
Functional oral intake scale	5.0(3.0-6.0)	6.0(3.5-6.0)	0.370
Mini-nutritional assessment	18.5(14.5-21.0)	18.0(15.1-18.9)	0.402
**PAC community hospitals** (%)			
Improved functional numbers, median (IQR)	6.0(5.0-8.0)	3.5(1.0-6.0)	<0.001
Improved functional numbers (≥5 vs. <5)	203(82.2)	7(35.0)	<0.001
Improved funtionality			
MRS	139(56.3)	4(20.0)	0.004

ADLs[f]	218(88.3)	9(45.0)	<0.001
IADLs	129(52.2)	5(25.0)	0.043
FOIS	100(40.5)	8(40.0)	1.000
MNA	162(65.6)	9(45.0)	0.177
BBS[f]	219(88.7)	13(65.0)	0.006
FMA-modified sensation	120(48.6)	8(40.0)	0.728
FMA-motor[f]	192(77.7)	13(65.0)	0.240
MMSE[f]	192(77.7)	9(45.0)	0.008
CCAT[f]	103(41.7)	4(20.0)	0.317
EQ-5D-3L			
Mobility	103(41.7)	2(10.0)	0.020
Self-care	106(42.9)	2(10.0)	0.016
Usual activities	95(38.5)	3(15.0)	0.109
Pain/discomfort[f]	70(28.3)	3(15.0)	0.414
Anxiety/depression[f]	64(25.9)	1(5.0)	0.083

Continuous data were expressed as median (IQR, interquartile range) and analyzed by the Mann-Whitney *U* test. Categorical data were expressed as number and percentage and analyzed by the Chi-Square test. Abbreviations: BMI, body mass index; COPD, chronic obstructive pulmonary disease; ACCI, age-adjusted Charlson Comorbidity Index; Hba1c, glycated hemoglobin; eGFR, estimated glomerular filtration rate; NT-proBNP, N-terminal pro-B-type natriuretic peptide; ADLs, activities of daily living, IADLs, instrumental activities of daily living; PAC, post-acute care; MRS, Modified Rankin Scale; FOIS, functional oral intake scale; MNA, mini-nutritional assessment; BBS, Berg balance scale; FMA-modified sensation, range of motion of joints (0 to 44) of the Fugl-Meyer assessment; FMA-motor, upper extremity motor subscore (0 to 66) of the Fugl-Meyer assessment; MMSE, mini-mental state examination; CCAT, Concise Chinese Aphasia Test; EQ-5D-3L, 3-level 5-dimensional Euro-Quality of Life tool; eGFR, calculated by using modified Modification diet of renal disease (MDRD) formula, was utilized to evaluate renal function. [f]Fisher's exact test.

Supplementary Table 3 Secondary outcome in patients with cerebrovascular events

	**No (*n* = 200)**	**Yes (*n* = 62)**	***p* value**
**Cerebrovascular accident**			0.630
Ischemia (%)	165(82.5)	54(87.1)	
Hemorrhage (%)	35(17.5)	8(12.9)	
**Demographic profile**			
Age, median (IQR, years)	67.0(57.0-79.0)	68.0(59.0-80.0)	0.770
Gender, male (%)	137(68.5)	33(53.2)	0.040
Smoking (%)	68(34.0)	17(27.4)	0.417
Alcohol, No. (%)[f]	37(18.5)	10(16.1)	0.850
BMI, median (IQR, kg/m^2)	24.3(22.0-27.1)	24.3(22.3-28.0)	0.627
**Comorbidity profile** (%)			
Diabetes Mellitus	76(38.0)	19(30.6)	0.367
Hypertension	167(83.5)	53(85.5)	0.862
Hyperlipidemia	114(57.0)	44(71.0)	0.069
Cardiovascular disease	81(40.5)	24(38.7)	0.918
COPD[f]	5(2.5)	2(3.2)	0.671
ACCI, median (IQR)	4.0(3.0-6.0)	4.0(3.0-5.0)	0.260
**Laboratory profile**, median (IQR)			
Low-density lipoprotein cholesterol (mg/dL)	100.0(84.3-125.0)	106.5(89.3-131.5)	0.178
Fasting glucose (mg/dL)	123.0(105.3-161.0)	120.0(103.8-137.5)	0.254
Hba1c (%)	6.0(5.7-6.6)	5.9(5.5-6.6)	0.224
Albumin (g/dL)	3.9(3.6-4.1)	3.8(3.6-4.1)	0.561
eGFR (ml/min per $1.73m^2$)	82.8(60.8-103.3)	82.5(62.3-103.6)	0.813
Urine protien / creatinine ratio (mg/g)	0.1(0.1-0.3)	0.1(0.1-0.3)	0.444
NT-proBNP (pg/mL)	1999.0(429.0-6738.0)	482.7(114.2-1300.0)	0.012
**Assessment in General Hospital** (%)			
Modified rankin scale	4.0(3.0-4.0)	4.0(4.0-4.0)	0.001
ADLs	40.0(21.3-55.0)	25.0(15.0-40.0)	0.001
IADLs	1.0(0.0-2.0)	0.0(0.0-1.0)	0.017
Functional oral intake scale	6.0(3.3-6.0)	5.0(2.0-6.0)	0.015
Mini-nutritional assessment	18.5(15.5-21.5)	16.5(13.0-19.8)	0.002
**PAC community hospitals** (%)			
Improved functional numbers, median (IQR)	6.0(4.0-7.0)	7.0(6.0-8.0)	<0.001
Improved functional numbers (≥7 vs. <7)	70(35.0)	44(71.0)	<0.001
Improved funtionality			
MRS	108(54.0)	34(54.8)	1.000

ADLs	162(81.0)	60(96.8)	0.005
IADLs	98(49.0)	34(54.8)	0.460
FOIS	68(34.0)	38(61.3)	<0.001
MNA	118(59.0)	52(83.9)	0.001
BBS	171(85.5)	56(90.3)	0.497
FMA-modified sensation	91(45.5)	36(58.1)	0.149
FMA-motor	147(73.5)	55(88.7)	0.028
MMSE	147(73.5)	54(87.1)	0.045
CCAT	75(37.5)	31(50.0)	0.257
EQ-5D-3L			
Mobility	77(38.5)	26(41.9)	0.647
Self-care	76(38.0)	33(53.2)	0.059
Usual activities	70(35.0)	27(43.5)	0.260
Pain/discomfort	51(25.5)	21(33.9)	0.232
Anxiety/depression	37(18.5)	26(41.9)	<0.001

Continuous data were expressed as median (IQR, interquartile range) and analyzed by the Mann-Whitney *U* test. Categorical data were expressed as number and percentage and analyzed by the Chi-Square test. Abbreviations: BMI, body mass index; COPD, chronic obstructive pulmonary disease; ACCI, age-adjusted Charlson Comorbidity Index; Hba1c, glycated hemoglobin; eGFR, estimated glomerular filtration rate; NT-proBNP, N-terminal pro-B-type natriuretic peptide; ADLs, activities of daily living, IADLs, instrumental activities of daily living; PAC, post-acute care; MRS, Modified Rankin Scale; FOIS, functional oral intake scale; MNA, mini-nutritional assessment; BBS, Berg balance scale; FMA-modified sensation, range of motion of joints (0 to 44) of the Fugl-Meyer assessment; FMA-motor, upper extremity motor subscore (0 to 66) of the Fugl-Meyer assessment; MMSE, mini-mental state examination; CCAT, Concise Chinese Aphasia Test; EQ-5D-3L, 3-level 5-dimensional Euro-Quality of Life tool; eGFR, calculated by using modified Modification diet of renal disease (MDRD) formula, was utilized to evaluate renal function. [f]Fisher's exact test.
